# Supplementary material for: Aberrant Expression of Androgen Receptor Associated with High Cancer Risk and Extrathyroidal Extension in Papillary Thyroid Carcinoma
Source: Cancers (Basel). 2020 Apr 29;12(5):1109. doi: 10.3390/cancers12051109 (PMC7281729; doi:10.3390/cancers12051109)
Supplement: Supplementary file 1 [file cancers-12-01109-s001.pdf]

Supplementary Materials and Methods:

Analysis of microarray data in GEO datasets

We had downloaded two microarray datasets from NCBI Gene Expression Omnibus (GEO) dataset, including GSE3678 and GSE35570. Among the GSE3678 dataset, we analyzed gene-expression data from datasets containing up to 7 PTC and 7 normal thyroid tissue to analyze the gene expression profiles (Suppleental figure1 A). Among the GSE35570 dataset, 45 normal and 32 tumor microarray chips were analysed (figure S1B). The raw data file of this array was downloaded and the value of AR were examed and analyzed. We inputted the microarray raw data into Partek software to analyze AR expression differences between PTC and normal thyroid.

Supplementary Figure:

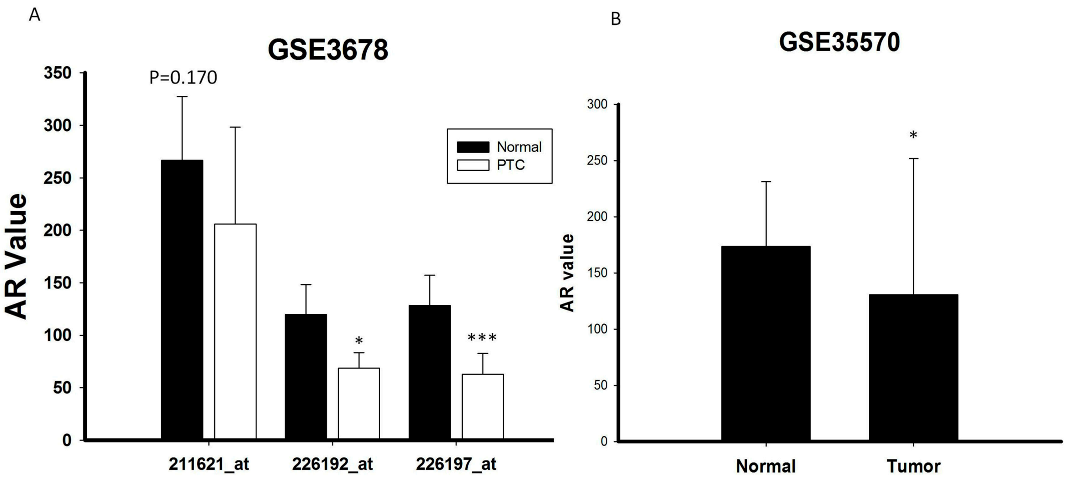

Figure S1: Low- expressed AR mRNA expression in PTC. The analysis of AR mRNA expression in open access GEO microarray. A. GSE3678 and B. GSE35570.

* $P<0.05$ and *** $P<0.001$ compared with the normal thyroid specimen.

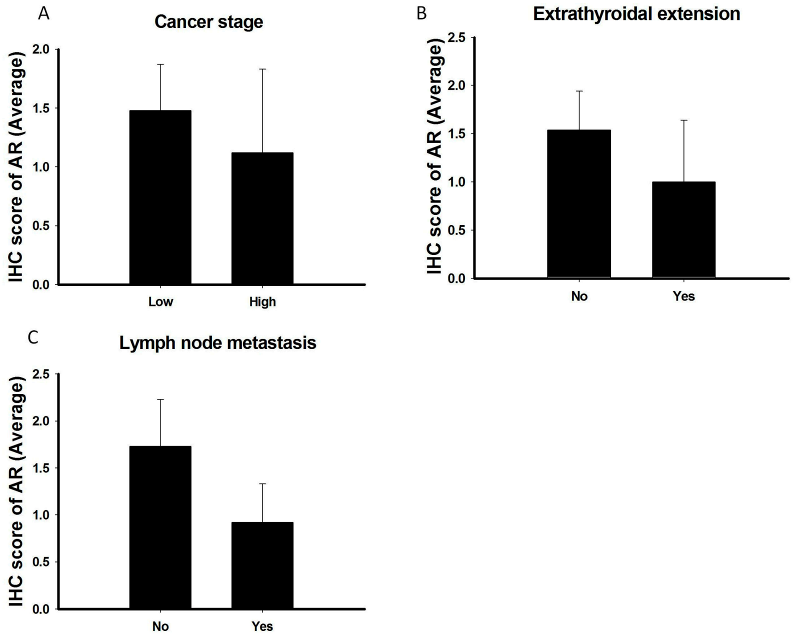

Figure S2. Association of AR protein expression with clinical features in PTCs. Analysis of AR IHC scoring with clinical features in 38 PTC patients stratified as follows: A) Cancer risk, low risk (stage I+II) v.s. High (stage III+IV) ; B) The presence of extrathyroidal extension and C) The presence of lymph node metastasis.

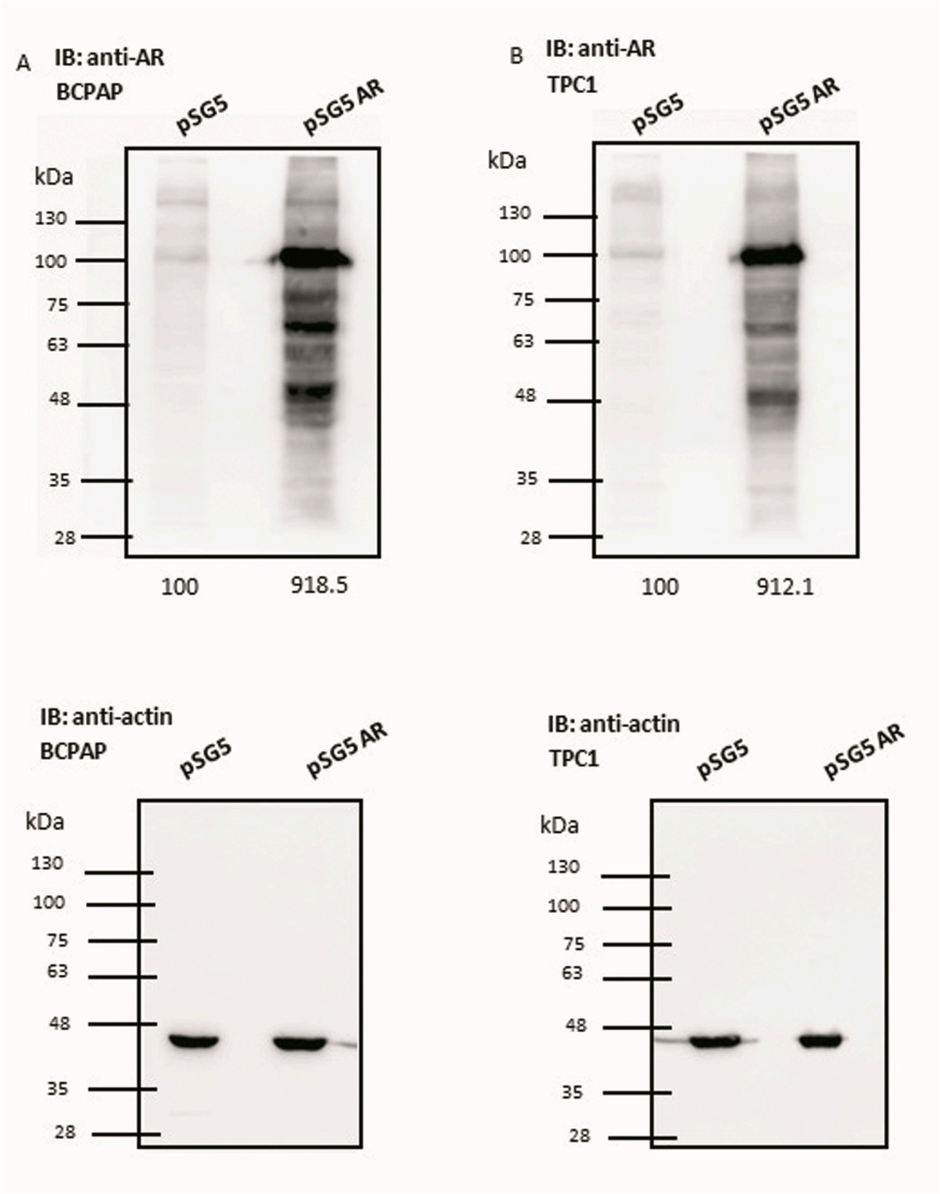

Figure S3. Western blot and densitometry analysis of protein expression levels of AR after transfection with pSG5-AR and pSG5 in A)BCPAP cells and B)TPC-1 cells.

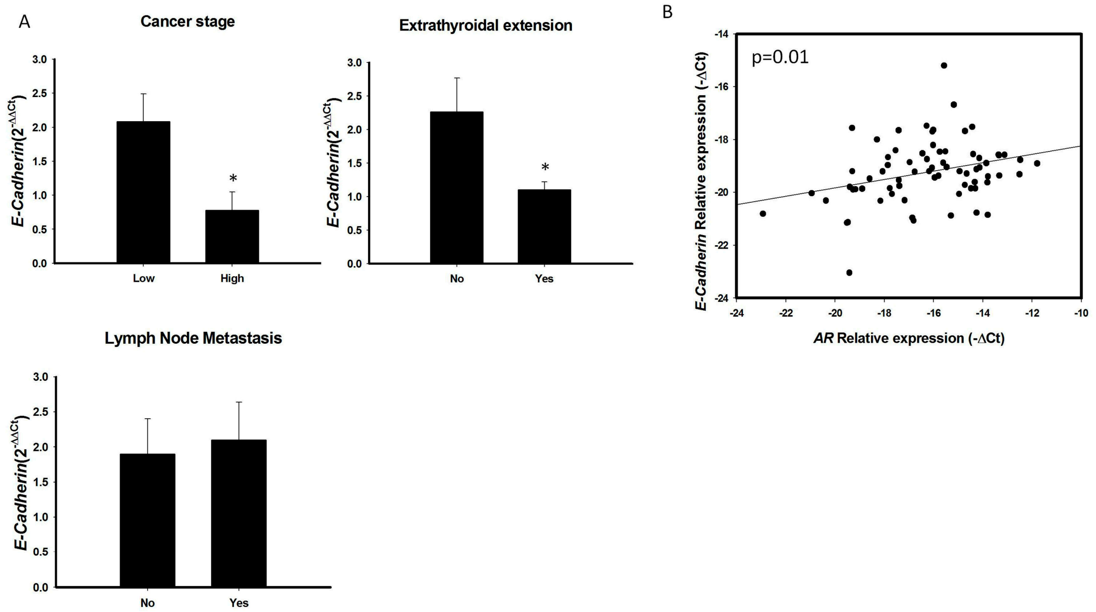

Figure S4. Association of *E-cadherin* mRNA expression with clinical features in PTCs. A) qRT-PCR analysis of *E-cadherin* mRNA with clinical features in 71 PTC patients stratified as follows: Cancer risk, low risk (stage I+II) v.s. High (stage III+IV); the presence of lymph node metastasis and the presence of extrathyroidal extension compared with the control. B) A scatter diagram shows that *AR* and *E-cadherin* expression levels are positively correlated ($P<0.05$).
